# Actigraphy-Based Associations Between Chronotype and Physical Activity in Older Adults

**DOI:** 10.1093/geroni/igab046.2878

**Published:** 2021-12-17

**Authors:** Hilary Hicks, Genna Losinski, Alexandra Laffer, Amber Watts

**Affiliations:** University of Kansas, Lawrence, Kansas, United States

## Abstract

Chronotype is a measure of the time of day people prefer to be most active or to sleep. There is a known relationship between chronotype and engagement in physical activity in young and middle-aged adults, such that individuals with a morning chronotype engage in more physical activity compared to those with an evening chronotype. Our study aimed to replicate this finding in an older adult sample. Actigraphy can be used to measure both physical activity and sleep. Because of its ability to capture information about bedtime and arise time, actigraphy can serve as an objective measurement of chronotype. Participants were 159 older adults (ages 60-89, M = 74.73) who wore an ActiGraph GT9X on their non-dominant wrist for 7 days in a free-living environment. Chronotype was measured continuously using the midpoint of the ActiGraph-calculated sleep interval. We used multiple regression to determine the relationship between physical activity and chronotype adjusting for sex, age, and body mass index. Results suggest that while these variables explain a significant amount of variance in physical activity, R2 = 19.0%, F (4, 152) = 8.921, p < .001, there is no significant relationship between chronotype and total physical activity in our sample, ß= -.117, p = .114. These findings are inconsistent with what has been shown in younger samples and suggest that the relationship between chronotype and physical activity may change as one ages. Future research should consider whether particular physical activity intensities (vs. total activity) may have a relationship with chronotype in older adults.

